# BreCaHAD: a dataset for breast cancer histopathological annotation and diagnosis

**DOI:** 10.1186/s13104-019-4121-7

**Published:** 2019-02-12

**Authors:** Alper Aksac, Douglas J. Demetrick, Tansel Ozyer, Reda Alhajj

**Affiliations:** 1 Department of Computer Science, University of Calgary, Calgary, AB T2N 1N4 Canada; 2 Department of Pathology & Laboratory Medicine, University of Calgary and Calgary Laboratory Services, Calgary, AB T2L 2K8 Canada; 3 Department of Computer Science, TOBB University of Economics and Technology, Ankara, 06510 Turkey; 4 Department of Computer Engineering, Istanbul Medipol University, Istanbul, Turkey

**Keywords:** Breast cancer, Histopathology, H&E staining, Annotation, Nottingham histologic score, Dataset

## Abstract

**Objectives:**

Histopathological tissue analysis by a pathologist determines the diagnosis and prognosis of most tumors, such as breast cancer. To estimate the aggressiveness of cancer, a pathologist evaluates the microscopic appearance of a biopsied tissue sample based on morphological features which have been correlated with patient outcome.

**Data description:**

This paper introduces a dataset of 162 breast cancer histopathology images, namely the breast cancer histopathological annotation and diagnosis dataset (BreCaHAD) which allows researchers to optimize and evaluate the usefulness of their proposed methods. The dataset includes various malignant cases. The task associated with this dataset is to automatically classify histological structures in these hematoxylin and eosin (H&E) stained images into six classes, namely mitosis, apoptosis, tumor nuclei, non-tumor nuclei, tubule, and non-tubule. By providing this dataset to the biomedical imaging community, we hope to encourage researchers in computer vision, machine learning and medical fields to contribute and develop methods/tools for automatic detection and diagnosis of cancerous regions in breast cancer histology images.

## Objective

Histopathological tissue analysis by a pathologist plays an important role in the diagnosis and prognosis of many types of cancer, such as breast. Staging and grading systems may vary for different types of cancer. Breast cancer is one of the most common types of cancer; it has its own grading systems. Nottingham grading system (also called the Elston-Ellis [1] modification of Scarff-Bloom-Richardson [2] grading system) is widely used criteria for the grade of breast tissues based on three main features, namely nuclear pleomorphism, tubular formation, and mitotic count, each of which is given 1 to 3 points. The scores of these three features are added together to determine an overall final score (in the range of 3–9) and the grade of the breast cancer. However, manually spotting and annotating the affected area(s) on histopathology images with high accuracy is regarded as the gold standard in cancer diagnosis and grading, but it is also a time-consuming and tedious task that requires considerable effort, expertise and experience of pathologists. These skills are mostly gained over time by analyzing more cases. Whereas this visual interpretation has strict guidelines, it brings a certain subjectivity to the histological analysis, and therefore leads to inter/intra-observer variability [3, 4] and some reproducibility issues. Besides, these issues may have a direct effect on patient prognosis and treatment planning. These problems can be alleviated by developing automated image analysis tools in digitized histopathology. Thanks to the rapid development in the image capturing and analysis technology which could be employed to not only give more insight to but also guide pathologists in detecting and grading infected cases. These quantitative computational tools aim to improve the quality of pathology researchers concerning speed and accuracy.

Thus, it is imperative to develop an automatic assessment tool for the quantitative and qualitative analysis in order to help in removing this drawback. However, histopathological examination of tissues is still a challenging problem since fixation, embedding, sectioning and staining steps in tissue preparation produce large amounts of artifacts and differences [5]. Besides, the variability in size, shape, location, texture of nuclei turn automated detection into a tedious and more difficult task. We believe that our various annotations from different cases will help to provide good enough information about these challenging situations.

## Data description

In this paper, we present a dataset of breast cancer histopathology images named BreCaHAD (Table 1, Data set 1) which is publicly available to the biomedical imaging community [6]. The images were obtained from archived surgical pathology example cases which have been archived for teaching purposes. Nottingham Grading System is an international grading system for breast cancer recommended by the World Health Organization, where the assessment of three morphological features (tubule formation, nuclear pleomorphism, and mitotic count) is used for scoring to decide on the final grade of the cancer case. To get these features, the H&E stained histological images are annotated or marked by a pathologist as either mitosis, apoptosis, tumor nuclei, non-tumor nuclei, tubule, and non-tubule. The sample cases are collected from various scenarios ranging from histological structures with clear boundaries to poorly differentiated structures with lack of typical features.

**Table 1:** Overview of data files/data sets

Label	Name of data file/data set	File types (file extension)	Data repository and identifier (DOI or accession number)
Data file 1	annotation_details.xlsx	MS Excel file (.xlsx)	Figshare (10.6084/m9.figshare.7379186)
Data file 2	original.png	Image file (.png)	Figshare (10.6084/m9.figshare.7379186)
Data file 3	annotated.png	Image file (.png)	Figshare (10.6084/m9.figshare.7379186)
Data file 4	data.json	JSON format file (.json)	Figshare (10.6084/m9.figshare.7379186)
Data set 1	BreCaHAD.zip	Archive file (.zip) containing dataset	Figshare (10.6084/m9.figshare.7379186)

The BreCaHAD dataset contains microscopic biopsy images which are saved in uncompressed (.TIFF) image format, three-channel RGB with 8-bit depth in each channel, and the dimension is 1360 × 1024 pixels and each image is annotated (see Table 1, Data file 2–3). These annotations are mitosis, apoptosis, tumor nuclei, non-tumor nuclei, tubule, and non-tubule. They are used in the assessment of three morphological features, namely nuclear pleomorphism, tubular formation, and mitotic count. Besides, breast tissue biopsy slides are used to generate samples is stained with hematoxylin and eosin (H&E). The same acquisition conditions and settings are used to obtain digitized images from tissue sample slides with a 0.514 µm × 0.527 µm per pixel at 40×, the camera at 40× objective captures 700 microns by 540 microns of microscopic image with a chip of 1360 × 1024 pixels. The images were captured under brightfield illumination with a Zeiss 40× oil objective on a Ziess Axiophot microscope through a 10× magnifier to a Spot Pursuit PR3440 camera controlled by Spot v5.2 software. While an automatic exposure mode is selected for the camera, the focusing is done manually for each slide.

All specimens were breast tissue fixed in 10% neutral buffered formalin (pH 7.4) for 12 h, processed in graded ethanol/xylene to Surgiplast paraffin. All sections were cut at 4 microns thickness, deparaffinized and stained with Harris’ hematoxylin and 1% eosin as per standard procedures. Specimens have been archived from 2 to 20 years, hence slight differences in staining and color characteristics reflect the procedures and reagents used over time. The dataset currently contains four malignant tumors (breast cancer): ductal carcinoma (DC), lobular carcinoma (LC), mucinous carcinoma (MC), and tubular carcinoma (TC). The distribution of annotations in the previously mentioned six classes and the format of the annotations for the BreCaHAD dataset can be found in Table 1, Data file 1.

The annotations for the BreCaHAD dataset are provided in JSON (JavaScript Object Notation) format. In the given Table 1, Data file 4, the JSON file (ground truth) contains two mitosis and only one tumor nuclei annotations. Here, *x* and *y* are the coordinates of the centroid of the annotated object, and the values are between [0, 1] (divided by width and height of an image).

By providing this dataset for research purposes, we wish to promote research in computer-aided diagnosis for breast cancer histopathology. Thus, researchers can optimize and prove the usefulness of their proposed methods while experimenting with this dataset.

## Limitations

The limited pixel/image tonal range of the images due to the camera, slight differences in color due to differing batches of hematoxylin over time, and the optical resolution of the 100× oil objective and immersion oil medium as these images were meant to reflect actual surgical pathology images typically used by diagnostic surgical pathologists to evaluate breast biopsies. In addition, the overall grading score for each case is not available and also the classification label is not included as either ductal carcinoma, lobular carcinoma, mucinous carcinoma or tubular carcinoma for each image.

## Abbreviations

BreCaHAD: breast cancer histopathological annotation and diagnosis dataset
H&E: Hematoxylin and Eosin
DC: ductal Carcinoma
LC: lobular Carcinoma
MC: mucinous Carcinoma
TC: tubular Carcinoma
JSON: JavaScript Object Notation
